# Learning Where to Look for High Value Improves Decision Making Asymmetrically

**DOI:** 10.3389/fpsyg.2017.02000

**Published:** 2017-11-15

**Authors:** Jaron T. Colas, Joy Lu

**Affiliations:** ^1^Computation and Neural Systems Program, California Institute of Technology, Pasadena, CA, United States; ^2^The Wharton School, University of Pennsylvania, Philadelphia, PA, United States

**Keywords:** decision making, reward learning, value, attention, visual orienting, oculomotor control, spatial processing, eye-tracking

## Abstract

Decision making in any brain is imperfect and costly in terms of time and energy. Operating under such constraints, an organism could be in a position to improve performance if an opportunity arose to exploit informative patterns in the environment being searched. Such an improvement of performance could entail both faster and more accurate (i.e., reward-maximizing) decisions. The present study investigated the extent to which human participants could learn to take advantage of immediate patterns in the spatial arrangement of serially presented foods such that a region of space would consistently be associated with greater subjective value. Eye movements leading up to choices demonstrated rapidly induced biases in the selective allocation of visual fixation and attention that were accompanied by both faster and more accurate choices of desired goods as implicit learning occurred. However, for the control condition with its spatially balanced reward environment, these subjects exhibited preexisting lateralized biases for eye and hand movements (i.e., leftward and rightward, respectively) that could act in opposition not only to each other but also to the orienting biases elicited by the experimental manipulation, producing an asymmetry between the left and right hemifields with respect to performance. Potentially owing at least in part to learned cultural conventions (e.g., reading from left to right), the findings herein particularly revealed an intrinsic leftward bias underlying initial saccades in the midst of more immediate feedback-directed processes for which spatial biases can be learned flexibly to optimize oculomotor and manual control in value-based decision making. The present study thus replicates general findings of learned attentional biases in a novel context with inherently rewarding stimuli and goes on to further elucidate the interactions between endogenous and exogenous biases.

## Introduction

**FIGURE 1:**
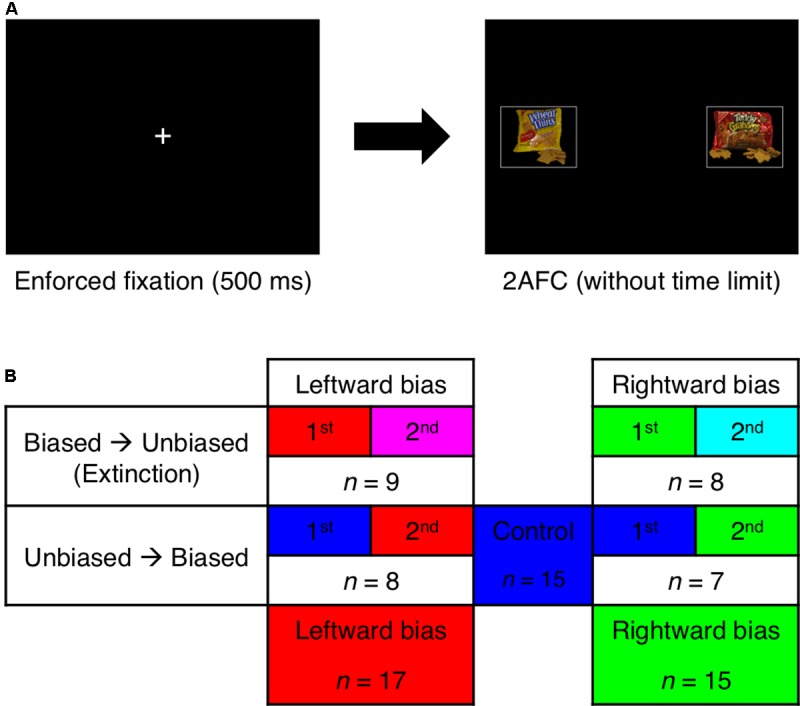
Paradigm. **(A)** Following mandatory fixation at the center of the display, the subject made a two-alternative forced choice (2AFC) between foods presented to the left and right while eye movements were monitored. **(B)** The stimulus with greater value was usually presented on the left side of the display for the leftward-bias condition (red) and usually presented on the right side of the display for the rightward-bias condition (green). Per a 2 × 2 between-subjects factorial design, the biased block of trials featuring this manipulation appeared either before or after an unbiased block with spatially balanced values. The pooled control condition (blue) was derived from the unbiased blocks that occurred first for half of the subjects. Unbiased blocks that occurred second in the sequence were set aside as the left-extinction (magenta) and right-extinction (cyan) conditions.

Regardless of whether the task is foraging in the wild or shopping in a modern store, there is often consistency in the spatial layout of one’s surroundings that could potentially be of use to the individual making decisions. Decision making is an active process that also entails searching for options and assessing what is actually available in order to compare the alternatives and select the best course of action. As this searching can demand precious time and effort, an organism’s optimal strategy in a stable environment would be to adjust the priors (i.e., in the Bayesian sense) initializing the information-seeking process in accordance with the patterned information content of previous observations. The work herein explored the possibility of such a strategy in visually guided (but manually executed) value-based decision making (**Figure 1A**), a typical setting in which the direction of one’s gaze functions as a proxy for the focus of selective attention. For visually minded animals such as humans, oculomotor control is especially representative of a directed sampling process that is driven by gains in information as well as gains in value—that is, minimization of uncertainty and maximization of reward, respectively (Hayhoe and Ballard, 2005; Tatler et al., 2011; Gottlieb, 2012; Gottlieb et al., 2014).

In a similar vein but within the domain of perceptual decision making, prior studies in psychophysics have reported learned biases of visuospatial attention in response to consistencies in the presentation of simple target stimuli that have been rewarded (e.g., Della Libera and Chelazzi, 2006, 2009; Liston and Stone, 2008; Hickey et al., 2010b, 2011; Krebs et al., 2010; Kristjánsson et al., 2010; Anderson et al., 2011a,b; Theeuwes and Belopolsky, 2012; Chelazzi et al., 2014; for review see Awh et al., 2012; Chelazzi et al., 2013; Anderson, 2016; Bourgeois et al., 2016). Furthermore, this line of research has begun to shed light on neurophysiological manifestations of such biases as yet further evidence (e.g., Kawagoe et al., 1998; Ikeda and Hikosaka, 2003; Hikosaka et al., 2006; Peck et al., 2009; Hickey et al., 2010a; Krebs et al., 2011; Yasuda et al., 2012; Kim and Hikosaka, 2013). With priming observed across various perceptual-discrimination tasks, task-relevant stimuli newly imbued with value elicit faster and more correct behavior. On the other hand, irrelevant stimuli that were previously associated with reward can still capture attention in extinction so as to instead interfere with performance in volatile environments when learned information is no longer applicable (Rutherford et al., 2010; Le Pelley et al., 2015; MacLean et al., 2016; Bucker and Theeuwes, 2017). This contrast illustrates how heterogeneous factors—whether internal or external and whether past or present—can be intertwined in proximal subdecisions about the deployment of attention (e.g., deciding where to look next), such that the traditional dichotomy of bottom–up and top–down (i.e., salience-driven and goal-directed, respectively) processes in attention can be blurred (Awh et al., 2012; Krauzlis et al., 2014). Yet, the scope of research on interactions between associative learning and attentional biases has heretofore been limited to perceptual decisions grounded in objective sensory features of stimuli rather than their subjective likeability.

The present study introduces a paradigm involving value-based decisions about complex stimuli (i.e., foods) that were made while eye movements were monitored in a structured setting more reminiscent of foraging or a modern analog such as shopping. Ecological relevance aside, the task stands apart in that one would only implicitly learn where to seek out the most valuable stimuli without having to learn which stimuli are valuable to begin with because a given food’s value is determined internally and subjectively. Of further interest is how inducing a spatial bias of attention would play out when robust biases are already present endogenously, as has been documented for tasks of this variety (Krajbich et al., 2010; Krajbich and Rangel, 2011; Reutskaja et al., 2011). Presumably due to some combination of not only innate biases (Vallortigara, 2006; Rugani et al., 2010; Frasnelli et al., 2012) but also deeply ingrained cultural conventions (e.g., reading from left to right) (Chokron and Imbert, 1993; Chokron and De Agostini, 1995; Chokron et al., 1998) that involve learning over much longer temporal scales, human subjects from our Westernized American population exhibit a striking predisposition to first examine the left side of a symmetric display. Thus, a key aspect of this experiment was that the manipulation attempted to bias the observer in either direction with repeated exposure to relatively more valuable goods at a single location (**Figure 1B**). As such, this design allowed for dissociation of the endogenous and exogenous forces that coalesce into orienting and choice behavior. Among the findings was a noteworthy asymmetry between learning to look to the left for high value and learning to look to the right for high value that also differentially affected the manually executed decisions.

## Materials and Methods

### Participants

Thirty-two (male:female = 16:16) of 35 volunteers between 18 and 35 years old from Caltech and the local community completed the study with proper acquisition of eye-tracking data. Criteria for participation included enjoying and regularly eating common American snack foods such as those used for the experiment. Participants provided informed written consent for a protocol approved by the California Institute of Technology Institutional Review Board. Participants were paid $20 for completing the study in addition to receiving chosen foods.

### Experimental Procedures

The subject first completed an ancillary rating task. Images of 100 generally appetitive snack foods were presented against a black background one at a time. For each trial, the subject was given unlimited time to rate the desirability of eating a given food at the end of the experiment according to a five-point Likert scale ranging from “strongly dislike” (1) to “strongly like” (5). The response was delivered by pressing the key corresponding to the selected number on a keyboard. These chromatic images had a resolution of 576 × 432 pixels and each subtended 25° × 19° of visual angle. The scale was displayed for reference above the food as black Arabic numerals on gray button icons below white text descriptors—altogether subtending 25° × 4°. The selected rating was highlighted on the scale with a white rectangle for 500 ms of feedback following the response. Trials were separated by an intertrial interval of 500 ms, during which only a white fixation cross was displayed centrally. The order of presentation was randomized for each subject. Stimuli were presented on a 15-inch LCD monitor with a resolution of 1024 × 768 pixels at a distance of 38 cm as part of an interface programmed using MATLAB and the Psychophysics Toolbox (Brainard, 1997).

A schematic of the two-alternative forced-choice (2AFC) task is shown in **Figure 1A**. The same images of foods were instead presented in pairs while the subject’s eye movements were recorded. Positions of both eyes were acquired at 50 Hz and converted to Cartesian coordinates for the screen in real time using a Tobii x50 desktop-mounted eye-tracking system. Trials were only initiated once the eye tracker’s algorithm verified during the intertrial interval that the subject’s direction of gaze had been stabilized for at least 500 ms on a white fixation cross subtending 0.8° × 0.8° at the center of the display. Upon removal of the fixation cross, the two stimuli were centered at eccentricities 15° to the left and right of the fixation point such that only one could be foveated at any given instant. The subject was given unlimited time to make a binary choice indicating which of the foods would be preferable to eat at the end of the experiment. The response was delivered by pressing one of two keys with either the left or the right index finger. The images were scaled down to 250 × 200 pixels and delineated by white rectangles each subtending 11° × 9°.

The pairings and their order were randomized for each subject with two constraints—the first being that absolute differences in subjective value were uniformly distributed across the set {1, 2, 3} according to each individual’s ratings; these were to correspond to high, medium, and low difficulty levels, respectively. The lowest difficulty level of 4 was excluded to limit redundancy. A second constraint related to the key experimental manipulation in this 2AFC task, which was divided into “biased” and “unbiased” blocks of 200 trials each. During the unbiased block, the stimulus with greater value was presented to either visual hemifield with equal probability. While the subject was not instructed about the possibility of such a manipulation, the biased block was instead characterized by the skewed appearance of greater value in either the left or the right hemifield for 90% of trials. According to a 2 × 2 between-subjects factorial design (**Figure 1B**), each subject was randomly assigned to one of four initial groups distinguished by the location where the bias was induced (i.e., leftward bias or rightward bias) and the counterbalanced ordering of the blocks (i.e., biased block before or after unbiased block).

The subject was required to refrain from eating or drinking anything except for water for at least 4 h prior to the experiment. The procedure was incentive-compatible (Hurwicz, 1972) inasmuch as the hungry subject was informed that one of the choices made was to be selected randomly and implemented at the end of the session. Upon completion, the subject was provided with this chosen food and required to remain within the laboratory for 15 min or until all of the item had been consumed.

### Data Analysis

Prior to the main analysis, data were first concatenated into three between-subject conditions (**Figure 1B**)—namely, leftward bias, rightward bias, and control. Biased blocks were combined across the two ordinal positions, whereas unbiased blocks were only recognized as belonging to the control condition if they occurred first and thus could establish an uncontaminated baseline. Unbiased blocks occurring second in the sequence were instead assigned to either the left-extinction condition or the right-extinction condition accordingly. Point estimates were generally limited to the latter 100 trials of each 200-trial block to assess effects after learning was shown to have occurred.

Eye-position data were analyzed with a standard region-of-interest (ROI) approach. Specifically, rectangular ROIs were first defined over the left and right stimulus locations, including symmetric extensions of 1° along each dimension to accommodate noisy data acquisition and microsaccades. Coordinates for the subject’s gaze were averaged across parallel streams of data for the two eyes whenever feasible. The onset of visual fixation was marked by the moment at which the subject’s direction of gaze first landed within either ROI. Fixation was coded as terminated once the gaze fell outside of that ROI if the gaze subsequently landed on the contralateral ROI. Fixation outside of either ROI both preceded and followed by fixation within a single ROI was coded as a single saccade to that ROI under the assumption that the intervening period merely reflected inevitable sources of data loss such as blinking.

For each condition, two aspects of eye movements were assessed and compared with respect to either spatial location or hedonic value. The former metric corresponded to the distribution of the first saccades at trial onset, whereas the latter corresponded to the differential allocation of dwell time across entire trials. Accompanying the mean across the latter half of a block in the presented results, centered moving averages were computed trialwise with a symmetric window of 21 trials to depict the time course of learning. The frequency of initial saccades to one side was compared with the chance level of 50% within each of the main learning conditions using one-tailed (or two-tailed in the case of the control condition) one-sample *t*-tests, and these frequencies were compared between conditions using one-tailed independent-samples *t*-tests. However, it should be noted that the assumption of wholly independent samples was overly stringent when comparing bias and control conditions with overlapping sets of subjects. In a similar vein, 95% confidence intervals as always provided are two-tailed in the interest of being conservative. Omitting the redundant control condition, similar tests were conducted for the frequency of initial saccades to whichever side contained the stimulus with greater value; however, a two-tailed test was used to compare the bias conditions. Analogous tests were conducted for the proportion of time within a trial that gaze was directed at either a fixed side or the side featuring greater value. It was only this very last set of tests that remained one-tailed for the extinction conditions, whereas two-tailed tests were employed otherwise in line with the more exploratory nature of these subsequent analyses.

Accuracy, which reflects the frequency of congruent choices of the option with greater value, was compared with the chance level of 50% within each condition and within each of three classifications of difficulty using one-tailed one-sample *t*-tests. Additionally of interest for the learning conditions were tests against the baseline performance level of 90% that could be achieved by heuristically choosing the more frequent response rather than properly performing the value-based task. Differences in accuracy between conditions were tested for using one-tailed independent-samples *t*-tests for comparisons between bias and control conditions along with a two-tailed test for comparing bias conditions. Each subject’s median reaction time (RT) was calculated separately for left- and right-option choices. RTs for each side were compared between pooled conditions using one-tailed independent-samples *t*-tests. As a complementary analysis, differences in RT between left and right choices were tested for within each condition using one-tailed (or two-tailed in the case of the control condition) one-sample *t*-tests, and these differences were additionally compared between conditions using one-tailed independent-samples *t*-tests.

## Results

### Learning: Eye Movements

As concerns eye movements, of primary interest were the options attended to first within each trial and the amount of time spent examining either option. Crucially, effects of habitual spatial biases would be intertwined with effects of hedonic value, which was encapsulated by ratings of how likeable each food would be. Analyses focused on the latter half of each block—after a point at which essential learning about the state of the environment was shown to have taken effect.

**FIGURE 2:**
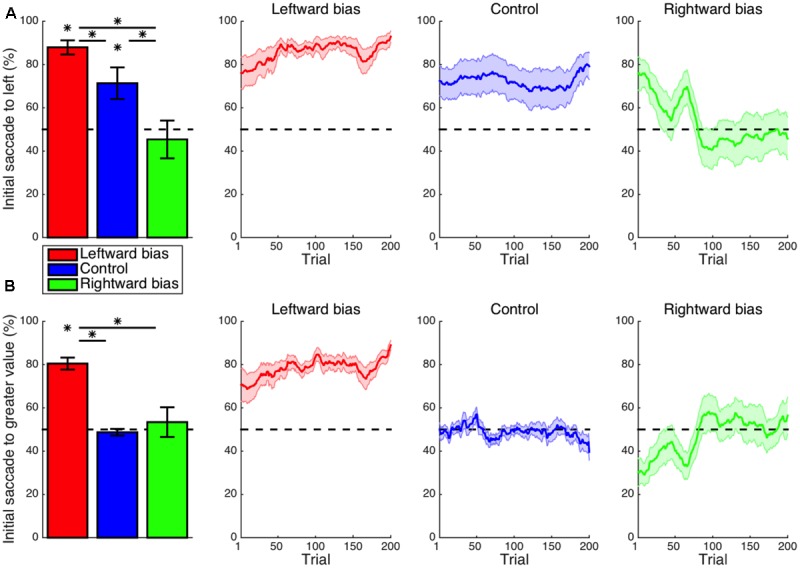
Learning: initial saccade. **(A)** Shown for each condition in the leftmost panel is the mean frequency of initial saccades to the stimulus presented to the left visual hemifield. The default leftward bias observed in the control condition (*p* < 0.05) was enhanced in the leftward-bias condition (*p* < 0.05) and neutralized in the rightward-bias condition (*p* < 0.05). Moving averages across trials are provided for reference as a depiction of the time courses of these effects during learning. Saturation of effects of learning was evident by halfway into the block of trials. **(B)** The frequency of initial saccades to the stimulus with greater value. As an exploitation of the experimental manipulation, first looking left in the leftward-bias condition corresponded to usually first looking at the stimulus with greater hedonic value (*p* < 0.05). Bar plots represent the latter half of a block. Error bars indicate standard errors of the means across subjects. Asterisks indicate statistical significance (*p* < 0.05).

Replicating previous reports of inherent leftward biases of visuospatial attention (Krajbich et al., 2010; Krajbich and Rangel, 2011; Reutskaja et al., 2011), the frequency of the first saccade within a trial being directed to the stimulus presented in the left visual hemifield (**Figure 2A**) was significantly greater than the chance level in the control condition (*M* = 21.3%, CI = [5.6, 37.1], *t_14_* = 2.91, *p* = 0.012). Whereas the control condition lacked any spatial pattern for subjective value, the bias conditions typically featured high-valued stimuli on one side of the display without the subject being explicitly instructed as to this arrangement. For the leftward-bias condition, initial saccades to the left were more frequent than expected by chance (*M* = 37.9%, CI = [31.0, 44.8], *t_16_* = 11.64, *p* < 10^-8^) and additionally more frequent as compared with the control condition (*M* = 16.6%, CI = [0.9, 32.3], *t_30_* = 2.15, *p* = 0.020). For the rightward-bias condition, however, the frequency of initial saccades to the right-side stimulus was not significantly greater than the chance level (*M* = 4.6%, CI = [-14.1, 23.4], *t_14_* = 0.53, *p* = 0.303) despite being significantly greater than the frequency observed in the control condition (*M* = 26.0%, CI = [-2.6, 49.3], *t_28_* = 2.28, *p* = 0.015). Juxtaposition of the leftward-bias and rightward-bias conditions thus revealed the first aspect of an asymmetry whereby a leftward bias at baseline was enhanced or neutralized, respectively. Even after learning had saturated within this timeframe, this default effect could not be overridden to a degree that would culminate in a reversed net-rightward bias.

As the signature manipulation of the experiment was that the option with superior value appeared in the same visual hemifield for nine out of every 10 trials, analogous analyses were instead conducted with regard to whichever side possessed greater value. The frequency of initial saccades to the stimulus with greater value (**Figure 2B**) was greater than the chance level for the leftward-bias condition (*M* = 30.4%, CI = [24.5, 36.3], *t_16_* = 10.94, *p* < 10^-8^)—an effect similarly exceeding that observed in the rightward-bias condition (*M* = 27.0%, CI = [12.5, 41.5], *t_30_* = 3.81, *p* < 10^-3^). The frequency of optimal initial saccades was not significantly greater than the chance level (*M* = 3.4%, CI = [-11.3, 18.2], *t_14_* = 0.50, *p* = 0.313) for the rightward-bias condition. Evident in the time course of learning, however, is that this apparent lack of an effect merely reflected the inability of a learned rightward bias to surpass the suddenly maladaptive intrinsic leftward bias despite fully neutralizing it. Altogether, the biases induced for initial saccades were consistent with selectively gathering information from loci with the greatest expected value as would be ideal.

**FIGURE 3:**
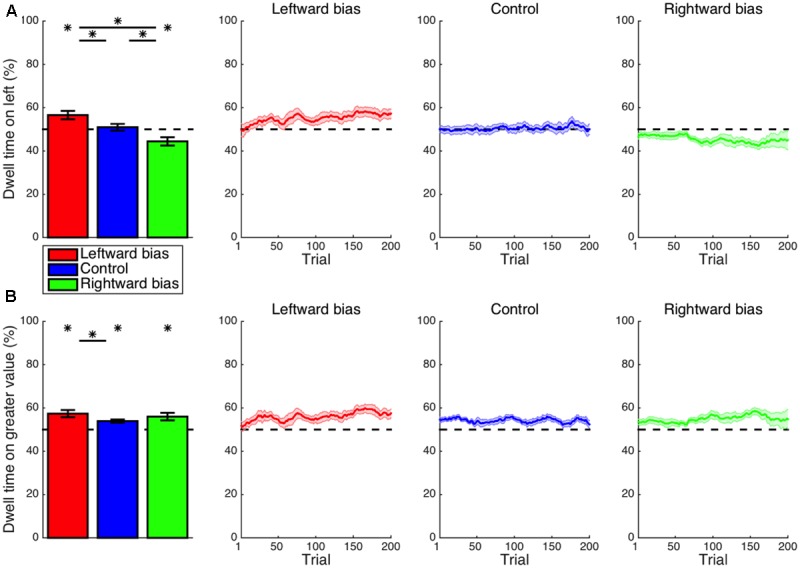
Learning: cumulative dwell time. **(A)** Shown for each condition is the mean proportion of time spent looking at the stimulus presented to the left side of the display throughout a trial. More time was spent fixating on the left-side stimulus for the leftward-bias condition (*p* < 0.05); likewise, more time was spent fixating on the right-side stimulus for the rightward-bias condition (*p* < 0.05). **(B)** The proportion of dwell time spent on the stimulus with greater value. Further asymmetry between conditions was revealed in that only the leftward-bias condition yielded longer dwell time at the location with greater value relative to control (*p* < 0.05). Asterisks indicate statistical significance (*p* < 0.05).

Expanding the scope of the analysis to the entire duration of a trial, the proportion of time spent fixating at the left location (**Figure 3A**) was not significantly different from the chance level for the control condition (*M* = 1.0%, CI = [-2.4, 4.3], *t_14_* = 0.62, *p* = 0.545), indicating that the aforementioned intrinsic leftward bias primarily affected only the beginning of an episode. For the leftward-bias condition, however, one’s gaze continued to be directed at the left-side stimulus for a significantly disproportionate amount of time (*M* = 6.6%, CI = [2.5, 10.6], *t_16_* = 3.40, *p* = 0.002). The rightward-bias condition was instead characterized by significantly more time dwelling on the right side (*M* = 5.6%, CI = [1.4, 9.7], *t_14_* = 2.89, *p* = 0.006). This overall pattern of effects resembled that found for the initial saccade in a manner suggesting that the same attentional biases permeate much of the temporal extent of decision making.

Again turning to the intersection of location and value, the proportion of time allocated to fixation on the stimulus with greater value (**Figure 3B**) was greater than the chance level even in the control condition (*M* = 3.9%, CI = [2.4, 5.5], *t_14_* = 5.50, *p* < 10^-4^). This was to be expected insofar as the spotlight of attention gravitates toward expected value so as to guide upcoming action selection (Shimojo et al., 2003; Simion and Shimojo, 2006, 2007; Krajbich et al., 2010, 2012; Krajbich and Rangel, 2011; Manohar and Husain, 2013; Towal et al., 2013). Yet, the disproportionate amount of dwell time on the more desirable alternative for the leftward-bias condition (*M* = 7.4%, CI = [3.8, 10.9], *t_16_* = 4.37, *p* < 10^-3^) further exceeded the control condition’s baseline (*M* = 3.4%, CI = [-0.5, 7.3], *t_30_* = 1.78, *p* = 0.042). In contrast, the disproportionate amount of dwell time on high value for the rightward-bias condition (*M* = 6.0%, CI = [2.3, 9.7], *t_14_* = 3.50, *p* = 0.002) was not significantly greater than the control level (*M* = 2.1%, CI = [-1.7, 5.9], *t_28_* = 1.11, *p* = 0.138). Yet, this proportion was not actually significantly greater for the leftward bias than for the rightward bias (*M* = 1.4%, CI = [-3.6, 6.3], *t_30_* = 0.56, *p* = 0.577). As a segue from the discovery that subjects were successful at optimizing oculomotor control as per the implicit statistics of the environment—albeit more robustly in the case of a leftward bias—the subsequent point of inquiry was to concern whether or not subjects were actually successful at optimizing their ultimate decisions with the benefit of more precisely deployed attention.

### Learning: Choices

Having established adaptive learning in eye movements, the accuracy of decisions and the speed with which they are made—namely, the RT—were expected to both improve to the extent that attending to preferable options would facilitate choosing them. That is, the influence of attentional modulation within a sequential-sampling process implies that selectively attending to an option biases decision making processes in favor of that option by means of a boost in the rate of accumulation of a decision signal. Such effects would impart the most direct evidence that the spatial statistics of the rewarding environment are not only being learned but also being exploited in harmony with what is prescribed for an agent with limited cognitive resources by normative decision theory.

**FIGURE 4:**
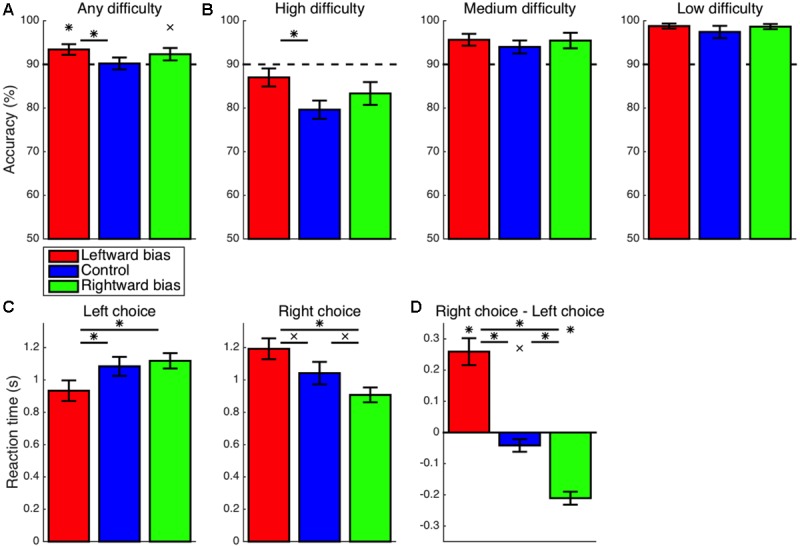
Learning: accuracy and reaction time (RT). **(A)** The overall accuracy of choices is depicted in relation to the baseline performance level of 90% set by the heuristic strategy of always choosing the more frequent response. Both the leftward-bias (*p* < 0.05) and rightward-bias (*p* < 0.07) conditions achieved even greater accuracy across all trials, albeit marginally so in the latter case. **(B)** Accuracy is shown separately for choices at each of the three levels of difficulty. At high difficulty with the most room for improvement, decision making was found to improve significantly relative to control for the leftward-bias condition (*p* < 0.05), which was also the condition yielding more robust effects on orienting. **(C)** RT is shown separately for left- and right-option choices, which were at least marginally faster in the leftward-bias (*p* < 0.05) and rightward-bias (*p* < 0.06) conditions, respectively, relative to the control condition. **(D)** Differences in RT between the two responses. Choices of the right option were marginally faster than choices of the left option in the control condition (*p* < 0.06). As expected, this baseline rightward bias was strengthened in the rightward-bias condition (*p* < 0.05) and reversed completely in the leftward-bias condition (*p* < 0.05). Crosses indicate marginal statistical significance (0.05 < *p* < 0.10). Asterisks indicate statistical significance (*p* < 0.05).

With regard to the accuracy of choices, the experimental manipulation allowed for 90% accuracy with recourse to the simpler heuristic strategy of invariably choosing the most frequent response (e.g., the left response in the leftward-bias condition). Nevertheless, accuracy across all trials at all three levels of difficulty (**Figure 4A**) exceeded this baseline level of 90% in both the leftward-bias condition (*M* = 3.4%, CI = [0.8, 6.0], *t_16_* = 2.81, *p* = 0.006) and the rightward-bias condition (*M* = 2.3%, CI = [-0.7, 5.4], *t_14_* = 1.65, *p* = 0.061), albeit marginally so in the latter case. These improvements in performance are evidence that, rather than relying upon speed-oriented heuristics, subjects continued to properly perform the value-based decision making task as they normally would but with the added benefit of learned biases. Furthermore, overall accuracy was greater for the leftward-bias condition than for the control condition (*M* = 3.2%, CI = [-0.5, 7.0], *t_30_* = 1.75, *p* = 0.045). In line with the previously reported asymmetries in effects on eye movements, this increase in accuracy relative to control was not significant for the rightward-bias condition (*M* = 2.1%, CI = [-1.9, 6.2], *t_28_* = 1.08, *p* = 0.145), but the difference between the leftward-bias and rightward-bias conditions was also non-significant (*M* = 1.1%, CI = [-2.7, 4.9], *t_30_* = 0.58, *p* = 0.566).

Choice accuracy was subsequently analyzed within bins assigned according to the difficulty of choices (**Figure 4B**). The most difficult trials, which correspond to the smallest differences in subjective value between stimuli, are of primary interest because these feature the most potential for improvement in performance as a consequence of learning. Accuracy was greater than the chance level even at high difficulty across all three conditions (*p* < 0.05), such that the critical tests probed differences between conditions. For trials of low or moderate difficulty, accuracy was saturated at near-ceiling levels, which precluded any significant differences between bias and control conditions among the four comparisons—namely, leftward bias at low difficulty (*M* = 1.4%, CI = [-1.6, 4.3], *t_30_* = 0.93, *p* = 0.180), rightward bias at low difficulty (*M* = 1.2%, CI = [-2.0, 4.4], *t_27_* = 0.79, *p* = 0.219), leftward bias at medium difficulty (*M* = 1.7%, CI = [-2.5, 5.8], *t_30_* = 0.82, *p* = 0.210), and rightward bias at medium difficulty (*M* = 1.5%, CI = [-3.3, 6.2], *t_28_* = 0.63, *p* = 0.265). However, the accuracy of noisier high-difficulty choices was greater in the leftward-bias condition than in the control condition (*M* = 7.4%, CI = [1.4, 13.4], *t_30_* = 2.51, *p* = 0.009). A non-significant effect was observed for the rightward-bias condition (*M* = 3.7%, CI = [-3.1, 10.6], *t_28_* = 1.11, *p* = 0.138), but the difference in accuracy between the leftward-bias and rightward-bias conditions at high difficulty did not reach statistical significance (*M* = 3.7%, CI = [-3.0, 10.4], *t_30_* = 1.12, *p* = 0.272).

First considering only choices of the left-side option, RT (**Figure 4C**) was indeed faster for the leftward-bias condition as compared to the control condition (*M* = 150 ms, CI = [-28, 329], *t_30_* = 1.72, *p* = 0.048). On the other hand, right-choice RT was marginally slower for the leftward-bias condition than for the control condition (*M* = 150 ms, CI = [-43, 344], *t_30_* = 1.59, *p* = 0.062). Nevertheless, overall speed improved insofar as left-option choices were much more frequent by design. Conversely, in the rightward-bias condition, right-option choices were marginally faster as compared to the control condition (*M* = 135 ms, CI = [-36, 305], *t_28_* = 1.62, *p* = 0.058). Yet, left-choice RT was not significantly slower in the case of the rightward-bias condition relative to the control condition (*M* = 34 ms, CI = [-120, 189], *t_28_* = 0.46, *p* = 0.326).

Next, differences in RT between left- and right-option choices were tested for within each condition (**Figure 4D**). Among these predominantly right-handed subjects, responses were delivered marginally more quickly with the right button in the control condition (*M* = 42 ms, CI = [-2, 85], *t_14_* = 2.06, *p* = 0.059). This effect suggests an intrinsic rightward bias that influences hand movements in concert with the intrinsic leftward spatial bias driving eye movements and the zoom lens of attention. This baseline effect was reversed such that instead left-option choices were faster for the leftward-bias condition (*M* = 259 ms, CI = [168, 351], *t_16_* = 6.00, *p* < 10^-5^). Likewise, right-option choices were more rapid for the rightward-bias condition (*M* = 211 ms, CI = [166, 255], *t_14_* = 10.16, *p* < 10^-7^) and to a degree that exceeded the baseline effect for the control condition (*M* = 169 ms, CI = [110, 228], *t_28_* = 5.84, *p* < 10^-5^).

Taken together, the results thus far indicate that subjects within the spatially structured environments of the leftward-bias and rightward-bias conditions learned to optimize value-based decision making processes with respect to both precision and speed—but especially when the reward environment conformed to preexisting leftward biases.

### Extinction: Eye Movements

Having demonstrated with the main analysis that learning did in fact occur as expected, the next set of analyses set out to determine the extent of any residual effects of either experimental manipulation in a subsequent extinction block with spatially balanced values. In other words, the only distinguishing feature between an extinction condition and the control condition lies in hysteresis due to the internal state of the subject. These extinction conditions were for the most part analyzed in the same fashion as before, beginning with the first saccade of a trial.

**FIGURE 5:**
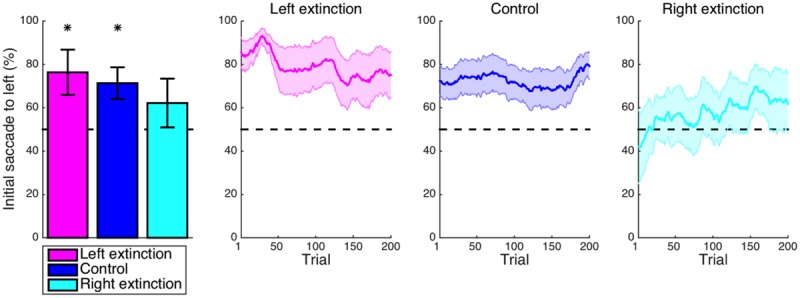
Extinction: initial saccade. A default leftward bias for the initial saccade as observed in the control condition (*p* < 0.05) was also found for the left-extinction condition (*p* < 0.05) but not the right-extinction condition (*p* > 0.05). Note that the plots that would correspond to those in **Figure 2B** are omitted here because of the absence of a spatial pattern for value in the extinction blocks, such that the subject was unable to predictively saccade to the stimulus with greater value by design (*p* > 0.05). Asterisks indicate statistical significance (*p* < 0.05).

Focusing first on the left-extinction condition, initial saccades to the left-hemifield stimulus (**Figure 5**) were still more frequent than expected by chance (*M* = 26.3%, CI = [2.3, 50.4], *t_8_* = 2.52, *p* = 0.036), but this effect was not significantly greater than the baseline effect observed in the control condition (*M* = 5.0%, CI = [-20.8, 30.8], *t_22_* = 0.40, *p* = 0.691). Although the respective leftward bias of the right-extinction condition was not significantly above chance (*M* = 12.2%, CI = [-14.4, 38.7], *t_7_* = 1.08, *p* = 0.314), it was not significantly lesser than the control level (*M* = 9.2%, CI = [-17.8, 36.1], *t_21_* = 0.71, *p* = 0.487), either. The pattern thus could align with an interpretation of at least to some extent returning to the baseline set by intrinsic biases in extinction.

**FIGURE 6:**
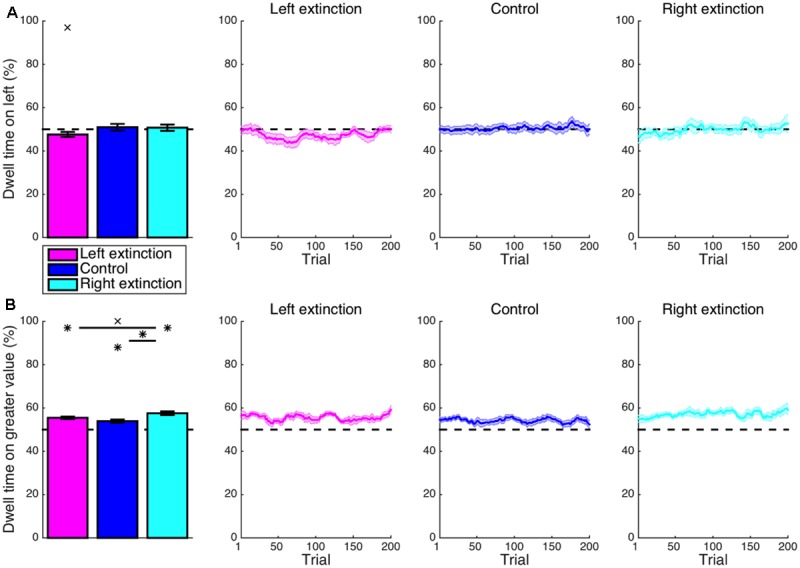
Extinction: cumulative dwell time. **(A)** Whereas the learned rightward bias in dwell time was neutralized for the right-extinction condition (*p* > 0.05), the respective leftward bias was even reversed by apparent overcompensation in the left-extinction condition such that there was actually a marginal rightward bias in dwell time (*p* < 0.08). **(B)** Only the right-extinction condition was characterized by longer dwell time at the location with greater value relative to control (*p* < 0.05). Crosses indicate marginal statistical significance (0.05 < *p* < 0.10). Asterisks indicate statistical significance (*p* < 0.05).

In contrast to the leftward bias in overall dwell time exhibited during learning, the left-extinction condition was characterized by apparent overcompensation such that a marginally disproportionate amount of time was actually spent fixating on the right side of the display (*M* = 2.4%, CI = [-0.3, 5.1], *t_8_* = 2.03, *p* = 0.077) (**Figure 6A**). Again, there was some lateralized asymmetry. Rather than being reversed, the learned rightward bias was neutralized in the right-extinction condition to produce a null leftward effect on dwell time (*M* = 0.7%, CI = [-2.7, 4.2], *t_7_* = 0.51, *p* = 0.629).

Although the proportion of time allocated to fixating on the stimulus with greater value (**Figure 6B**) was still well in excess of chance for the left-extinction condition (*M* = 5.5%, CI = [4.2, 6.8], *t_8_* = 9.69, *p* < 10^-5^), this imbalance was not significantly different from that observed in the control condition (*M* = 1.5%, CI = [-0.6, 3.7], *t_22_* = 1.49, *p* = 0.151). This value-based bias in dwell time was likewise significant for the right-extinction condition (*M* = 7.5%, CI = [5.6, 9.5], *t_7_* = 8.94, *p* < 10^-4^) and in this case even more robust than the biases exhibited in both the control (*M* = 3.6%, CI = [1.2, 6.0], *t_21_* = 3.11, *p* = 0.005) and left-extinction (*M* = 2.1%, CI = [0.0, 4.2], *t_15_* = 2.09, *p* = 0.054) conditions, albeit marginally so in the latter case. This improvement could reflect greater arousal as is fitting for a novel and uncertain environment coupled with the lack of a strong spatial bias as is fitting for a spatially balanced reward environment.

### Extinction: Choices

**FIGURE 7:**
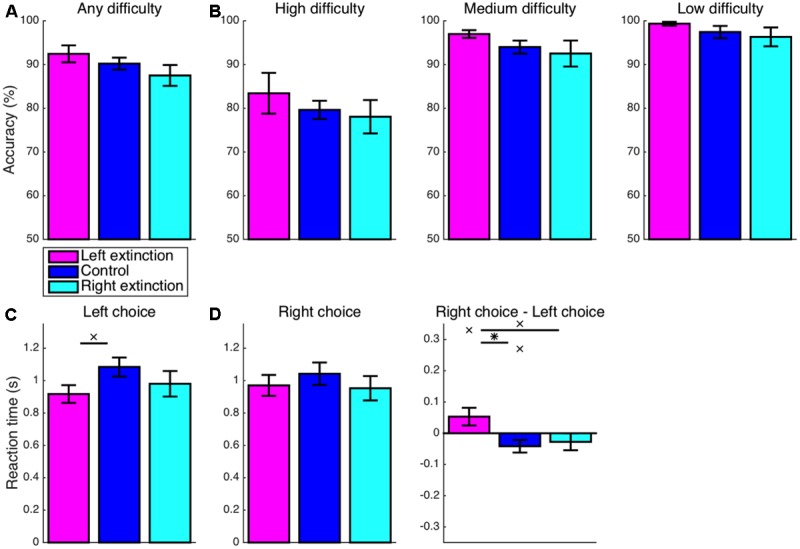
Extinction: accuracy and RT. **(A,B)** There were no significant differences with respect to accuracy for either of the extinction conditions (*p* > 0.05). **(C)** The RT was still marginally faster for left-option choices in the left-extinction condition relative to control (*p* < 0.07), but there was no longer a corresponding effect for right-option choices in the right-extinction condition (*p* > 0.05). **(D)** Contrary to the marginal rightward bias at baseline (*p* < 0.05), choices of the left option remained marginally faster than choices of the right option for the left-extinction condition (*p* < 0.10), whereas there was no corresponding rightward bias for the rightward-extinction condition (*p* > 0.05). Crosses indicate marginal statistical significance (0.05 < *p* < 0.10). Asterisks indicate statistical significance (*p* < 0.05).

Turning back to the accuracy of choices, this score was again significantly greater than the chance level for any combination of condition and difficulty (*p* < 0.05). Overall accuracy (**Figure 7A**) for the left-extinction condition was no longer significantly greater than the control level (*M* = 2.2%, CI = [-2.6, 7.1], *t_22_* = 0.96, *p* = 0.346). Likewise, any increase in accuracy relative to control in the left-extinction condition was non-significant specifically for trials of low (*M* = 1.9%, CI = [-2.0, 5.8], *t_22_* = 1.02, *p* = 0.317), medium (*M* = 3.0%, CI = [-1.3, 7.3], *t_22_* = 1.45, *p* = 0.161), and high (*M* = 3.8%, CI = [-5.5, 13.1], *t_22_* = 0.85, *p* = 0.406) difficulty (**Figure 7B**). Conversely, overall accuracy for the right-extinction condition was not significantly lesser than that observed in the control condition (*M* = 2.7%, CI = [-2.6, 8.0], *t_21_* = 1.05, *p* = 0.304). Furthermore, overall accuracy for the left-extinction condition did not fully surpass that for the right-extinction condition (*M* = 4.9%, CI = [-1.6, 11.4], *t_15_* = 1.62, *p* = 0.126). Any decrease in accuracy in the right-extinction was non-significant for low (*M* = 1.1%, CI = [-4.2, 6.4], *t_20_* = 0.44, *p* = 0.666), medium (*M* = 1.5%, CI = [-4.7, 7.6], *t_21_* = 0.49, *p* = 0.626), and high (*M* = 1.6%, CI = [-6.7, 9.8], *t_21_* = 0.39, *p* = 0.698) difficulty.

In keeping with the learned bias, the left-extinction condition was still characterized by marginally faster RT for left-option choices relative to control (*M* = 167 ms, CI = [-14, 347], *t_22_* = 1.91, *p* = 0.069) (**Figure 7C**). However, there was no corresponding effect for faster right-option choices in the right-extinction condition (*M* = 89 ms, CI = [-140, 319], *t_21_* = 0.81, *p* = 0.426). A corresponding asymmetric pattern applied to differences in RT between the two options (**Figure 7D**). As part of a significant deviation from the marginal rightward bias at baseline in the left-extinction condition (*M* = 95 ms, CI = [24, 165], *t_22_* = 2.78, *p* = 0.011), choices of the left option remained marginally faster than choices of the right option (*M* = 53 ms, CI = [-12, 118], *t_8_* = 1.88, *p* = 0.097). The right-extinction condition, on the other hand, did not produce a significant rightward bias in RT (*M* = 27 ms, CI = [-37, 91], *t_7_* = 1.01, *p* = 0.345).

Altogether, this latter set of findings concerning the extinction conditions suggests that oculomotor and manual biases as induced here can be unlearned in extinction relatively quickly.

## Discussion

All findings considered, this research has demonstrated the human brain’s capacity to learn where to look for maximal utility and thus make decisions more efficiently in a setting where spatial location and hedonic value are correlated despite no overt signs of such a correlation. Building upon related paradigms in psychophysics involving explicit, arbitrary designations of value to simple, abstract stimuli or locations (Awh et al., 2012; Chelazzi et al., 2013; Anderson, 2016; Bourgeois et al., 2016), this novel eye-tracking approach incorporated implicit learning of spatial attentional biases into value-based decision making with familiar, tangible stimuli (i.e., foods) that could be evaluated *a priori* independently of context or positions in space. To mitigate the susceptibility of noisy decision making processes to errors, subjects took into account the additional spatial information when available in accord with an optimal strategy. Rather than merely shifting the balance of the speed-accuracy tradeoff (Johnson, 1939) in favor of quickness via reliance upon heuristics (e.g., rapidly delivering the more frequent response without making an effort to evaluate and compare the alternative), the downstream effects of induced attentional biases successfully honed both speed and accuracy even in the absence of any time pressure other than that which is self-imposed.

A notable asymmetry distinguished the learning of a leftward attentional bias from the less robust learning of a rightward bias, reflecting conflict between the induced bias and an intrinsic leftward bias. The presence of a leftward bias replicated findings from similar studies in which Westernized American subjects (i.e., left-to-right readers) presented with visually symmetric alternatives have exhibited a proclivity for first scanning the left side of a display as well as its upper portion (Krajbich et al., 2010; Krajbich and Rangel, 2011; Reutskaja et al., 2011). The leftward aspect may reflect the more general, low-level phenomenon of left hemispatial overrepresentation implicated in tasks as basic as line bisection (Jewell and McCourt, 2000). Notwithstanding the innate right-hemispheric dominance of visuospatial attention in the human brain (de Schotten et al., 2011) and the abundance of innate leftward or left-to-right spatial biases in related forms of laterality throughout the animal kingdom (Vallortigara, 2006; Rugani et al., 2010; Frasnelli et al., 2012), however, the direction by which one scans the visual field is critical for these effects, such that right-to-left (e.g., Hebrew) readers instead naturally exhibit a contrary rightward bias as per divergent cultural norms (Chokron and Imbert, 1993; Chokron and De Agostini, 1995; Chokron et al., 1998). Further study of the current paradigm and others like it with human subjects molded by cultures that diverge with respect to these spatial biases will be necessary to fully explicate the relationships between immediate task-related biases learned over shorter temporal scales and sociocultural biases learned over longer temporal scales. That such asymmetry applies even for preferential decision making scenarios in which stimuli can be abstracted away from space, actions, and actual sensory properties altogether is remarkable for its implications vis-à-vis designing any sort of visual interface intended for human viewers (e.g., the layout of item labeling per Rebollar et al., 2015)—but especially for situations where the alternatives under consideration themselves map directly onto space.

Computational modeling that encompasses the dynamics of people’s preferential choices as well as the eye movements leading up to them has raised the importance of visual fixation and attention as part of an account of value-based decision making (Krajbich et al., 2010, 2012; Krajbich and Rangel, 2011; Towal et al., 2013). Although not applied directly here, such modeling forms the theoretical framework for the present study. This class of models emphasizes how attention-based mechanisms in general will selectively enhance the neural representation (i.e., signal-to-noise ratio) of an option (Yantis and Serences, 2003; Reynolds and Chelazzi, 2004; Maunsell and Treue, 2006; Cohen and Maunsell, 2009; Lim et al., 2011; McGinty et al., 2016; Leong et al., 2017) and, in doing so, ultimately bias decision signals being computed continuously by sequential-sampling processes. Although attention tends to at first be drawn to perceptually salient (Itti and Koch, 2001) or novel (Yang et al., 2009) stimuli (Desimone and Duncan, 1995), so too are gaze and its underlying attentional processes driven by the motivational salience (Schultz, 2015) or incentive salience (Robinson and Berridge, 1993) of options with greater value—and particularly so in the final moments prior to making a decision when acquisition of necessary information approaches its saturation point (Shimojo et al., 2003; Simion and Shimojo, 2006, 2007; Krajbich et al., 2010, 2012; Krajbich and Rangel, 2011; Manohar and Husain, 2013; Towal et al., 2013). Reflecting preferential looking (Fantz, 1961) and the mere-exposure effect (Zajonc, 1968) in parallel with information seeking, this cascade effect of gaze emerges as a positive-feedback loop is formed to the extent that attending to an option also makes it more likely to be chosen. Moreover, exogenous manipulation of eye movements and visual attention causally biases preferences in favor of specific options—whether via requirements for longer periods of exposure and visual fixation (Shimojo et al., 2003; Armel et al., 2008; Lim et al., 2011; Bird et al., 2012; Ito et al., 2014) or less directly via artificially increased perceptual salience (Milosavljevic et al., 2012).

The paradigm illustrated here essentially lies at the interface of associative learning and attention, two spheres of neural phenomena that hitherto have not been sufficiently linked in the literature of neuroscience and psychology—much less economics. As the findings herein have attested, attentional signals can be modulated by implicit learning even in naturalistic value-based decision making. Likewise, there is a firm theoretical basis for the notion that attention plays a critical role in selectively encoding the most relevant information into memory in the first place, raising yet further questions as to what extent different factors (e.g., reward or uncertainty) determine such relevance (Mackintosh, 1975; Underwood, 1976; Pearce and Hall, 1980; Dayan et al., 2000; Jiménez, 2003; Pearce and Mackintosh, 2010; Gottlieb, 2012; Le Pelley et al., 2016; Leong et al., 2017). Whereas effects on orienting as described here are entirely tractable within some variant of the basic reinforcement-learning framework (Rescorla and Wagner, 1972; Sutton and Barto, 1998)—and especially amenable to a temporal-difference algorithm (Sutton, 1988) given the continuous nature of events—the precise nature of the prediction-error signals or other feedback involved remains largely enigmatic. This set of issues adds a new dimension to the problem with computational modeling encompassing attention and eye movements in relation to not only decision making but also learning processes.

Setting aside goal-directed (i.e., model-based) learning (Tolman, 1948), the two-process theory of habitual (i.e., model-free) learning (Miller and Konorski, 1928; Rescorla and Solomon, 1967; Dayan and Balleine, 2002; O’Doherty et al., 2017) posits that instrumental (or operant) conditioning (Thorndike, 1898) is distinct from Pavlovian (or classical) conditioning (Pavlov, 1927), such that instrumental stimulus-response associations differ fundamentally from Pavlovian stimulus-stimulus associations. Within Pavlovian conditioning there is an additional division between preparatory and consummatory behaviors: the former are non-specific (e.g., autonomic arousal, pupil dilation), whereas the latter are responses specific to the stimulus type (e.g., orienting, approaching, salivating, chewing) (Konorski, 1967). In this context, an oculomotor orienting response is innate and reflexive while simultaneously possessing utility as a goal-directed action. As such, a biased response could feasibly be reinforced through either consummatory Pavlovian processes or instrumental processes. Further research will be necessary to determine the extent to which these effects of implicit learning on attention generalize beyond oculomotor control (e.g., to covert shifts of attention in the absence any motoric orienting), as this would be indicative of a broader and more flexible phenomenon of instrumental conditioning as opposed to a Pavlovian system embedded within oculomotor circuits. Along the same lines, another endeavor for future research will be to explore possible extraction of non-spatial features in learning how to optimally deploy attention—for example, relating asymmetry in value to contextual stimuli or time points within a sequence rather than spatial locations.

## Author Contributions

JL designed and conducted the experiment. JC analyzed the data and wrote the manuscript.

## Conflict of Interest Statement

The authors declare that the research was conducted in the absence of any commercial or financial relationships that could be construed as a potential conflict of interest.
